# Supplementary material for: Machine learning the electric field response of condensed phase systems using perturbed neural network potentials
Source: Nat Commun. 2024 Sep 18;15:8192. doi: 10.1038/s41467-024-52491-3 (PMC11411082; doi:10.1038/s41467-024-52491-3)
Supplement: Supplementary file 1 — Supplementary Information [file 41467_2024_52491_MOESM1_ESM.pdf]

Supplementary Information

# Machine learning the electric field response of condensed phase systems using perturbed neural network potentials

Kit Joll[1], Philipp Schienbein*[1,2], Kevin M. Rosso[3], and Jochen Blumberger†[1]

[1]Department of Physics and Astronomy and Thomas Young Centre, University College London, London, WC1E 6BT, United Kingdom

[2]Department of Physics, Imperial College London, Exhibition Rd, South Kensington, London, SW7 2AZ, United Kingdom

[3]Pacific Northwest National Laboratory, Richland, Washington 99354, United States

*to whom correspondence should be addressed, email: p.schienbein@ucl.ac.uk
†to whom correspondence should be addressed, email: j.blumberger@ucl.ac.uk

## Supplementary Note 1: Total energy and momentum conservation

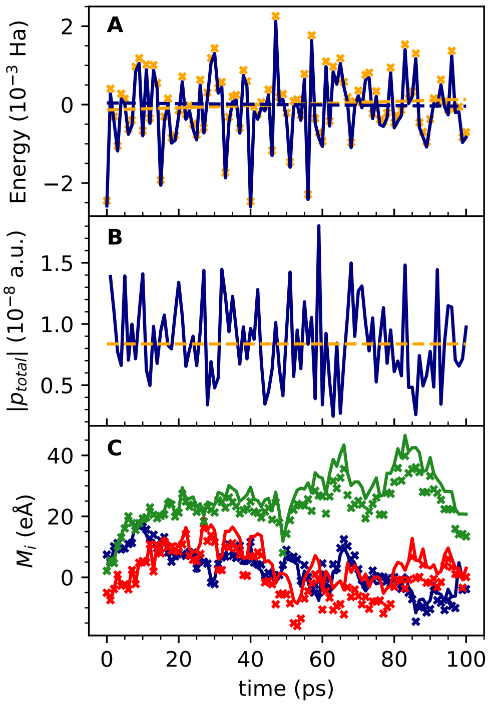


Figure S1: PNNP simulation of a box of 128 water molecules at a field strength of $0.0129\,\mathrm{V\,Å^{-1}}$. Panel A shows the total energy with its mean value shifted to zero as a function of simulation time. The field-induced contribution is calculated from the integrated time derivative of the polarization as predicted by APTNN (Eq. 5 main text, solid blue line) and from DFT reference calculations (yellow crosses). The dashed lines illustrate linear fits to that data using the same color code. Panel B shows the magnitude of the total momentum, displayed in atomic units, as a function of simulation time. Panel C presents the polarization as a function of simulation time, calculated by integration (Eq. 5 main text, solid lines) and from explicit DFT reference calculations (crosses) for the three spatial coordinatex $x$ (blue), $y$ (red), and $z$ (green). The electric field was applied along the $z$-direction. Source data are provided in the Source Data files.

## Supplementary Note 2: Electric field sweep

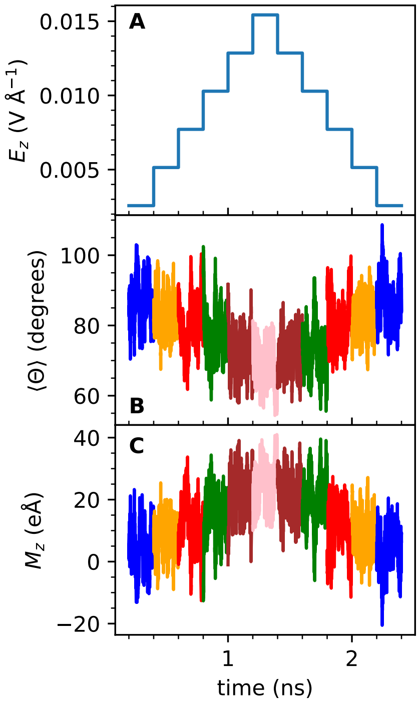


Figure S2: PNNP MD simulation of a field sweep going from 0.0026 V Å$^{-1}$ to 0.0154 V Å$^{-1}$, by increasing the applied field strengths by 0.0026 V Å$^{-1}$ every 200 ps (forward sweep) and reverting from 0.0154 V Å$^{-1}$ back to 0.0026 V Å$^{-1}$ by decreasing the applied field strengths by 0.0026 V Å$^{-1}$ every 200 ps (backward sweep). The applied field strength is depicted in panel A, while the response of the water molecules in terms of the average water orientation $\langle\Theta\rangle$ (see main text, Figure 2) and the polarization along the field ($M_z$) is shown in panels B and C, respectively. Source data are provided in the Source Data files.

## Supplementary Note 3: Convergence of dielectric constant

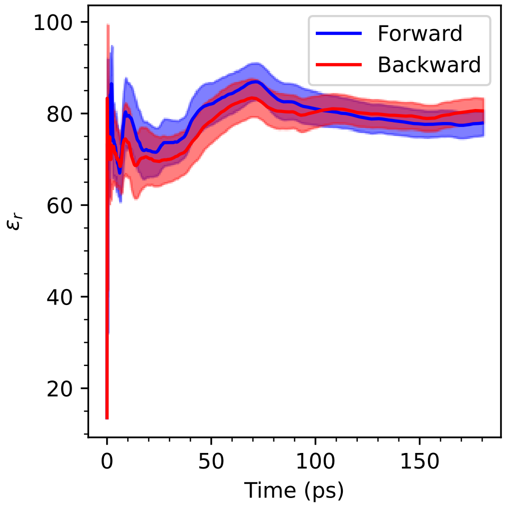


Figure S3: Convergence of the dielectric constant as a function of simulation time per electric field strength for the forward (blue) and backward (red) sweep, obtained from the data shown in Fig. S2. The dielectric constant was calculated from the change in mean polarization in response to the change in electric field, Eq. 7 main text. The shaded area illustrates the corresponding standard error of the slope obtained from the weighted linear fit to the polarization data at each time point. Source data are provided in the Source Data files.

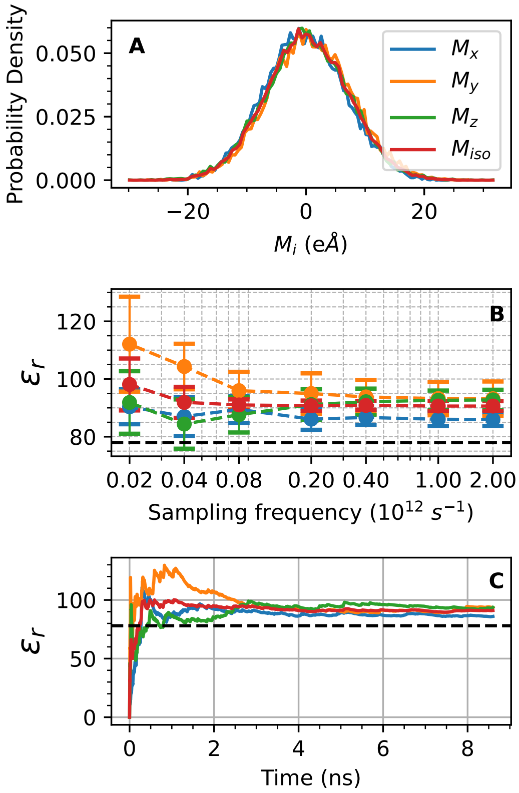


Figure S4: Convergence of the dielectric constant obtained from the mean square fluctuations of the dipole moment at zero field, Eq. 8 in the main text. Panel A depicts the distribution of the dipole moment for the different cartesian components and their average, $M_{\text{iso}}$, as obtained from a 8.6 ns c-NNP MD trajectory. The convergence of the dielectric constant is shown as a function of the frequency of DFT calculation of the total dipole moment along the 8.6 ns long trajectory (panel B) and as a function of the total simulation time when the total dipole moment is calculated at DFT level every 1 ps (panel C), corresponding to a frequency of $10^{12}$ $\text{s}^{-1}$ in panel B. The same colour code applies as in panel A. Source data are provided in the Source Data files.

## Supplementary Note 4: Relative RMSE

The relative RMSE for the atomic forces, displayed in Figure 1E in the main text is defined by

$$\overline{F}_{\mathrm{RMSE}} = \frac{F_{\mathrm{RMSE}}}{F_{\mathrm{RMS}}}$$

where

$$F_{\mathrm{RMSE}} = \sqrt{\frac{1}{3N_{\mathrm{a}}N_{\mathrm{c}}}\sum_{n}^{N_{\mathrm{c}}}\sum_{i}^{N_{\mathrm{a}}}\left(\mathbf{F}_{n,i}^{\mathrm{DFT}} - \mathbf{F}_{n,i}^{\mathrm{ML}}\right)^2}$$

and

$$F_{\mathrm{RMS}} = \sqrt{\frac{1}{3N_{\mathrm{a}}N_{\mathrm{c}}}\sum_{n}^{N_{\mathrm{c}}}\sum_{i}^{N_{\mathrm{a}}}\left(\mathbf{F}_{n,i}^{\mathrm{DFT}}\right)^2}.$$

$\mathbf{F}_{n,i}^{\mathrm{DFT}}$ is the force vector of atom $i$ in configuration $n$ calculated explicitly by DFT, $\mathbf{F}_{n,i}^{\mathrm{ML}}$ is the corresponding predicted force from the ML model, $N_{\mathrm{c}}$ and $N_{\mathrm{a}}$ are the number of configurations in the test set and the number of atoms in a configuration, respectively. Note that both the absolute and relative RMSE are caculated species-wise and then averaged. This definition of the relative error has been used previously in the machine learning molecular dynamics literature.[1] It relates the RMSE to the sum of force root-mean-square fluctuations and mean force. As elaborated in the main text, the relative RMSE is calculated separately for the total force, the unperturbed force contribution, and the field-induced force contribution.

## Supplementary Note 5: Comparison of PNNP with unpolarizable and polarizable force fields

In PNNP MD the magnitude of the force perturbation induced by the electric field (Fig. 1C main text) is much smaller than the magnitude of the unperturbed interatomic forces (Fig. 1B main text). This stimulates the question if a simpler point charge or flexible charge model could be used instead of the APTNN to achieve the same accuracy. Liquid water delicately depends on charge transfer and polarization effects which can impressively be illustrated with the help of its IR spectrum which can correctly be recovered by AIMD simulations at the level of DFT, see e.g. [2, 3, 4]. In stark contrast, if fixed point charges are employed (even on top of the same AIMD trajectories), some important features of the IR spectrum are missing [5]. It was even argued that non-polarizable force fields lead to incorrect vibrational couplings and thus artificial vibrational motion [5]. While Thole-type polarizable force fields were shown to model the structure and the dynamics of liquid water quite well, some inaccuracies were still identified when calculating vibrational spectra. [6] Remarkably, the IR spectrum is only fully recovered, if induced dipole moments, their correct geometry dependence, *and* charge transfer effects are explicitly accounted for by the model [6]. Although being supposedly simple, the APT explicitly includes all charge transfer and polarization effects. Consequently, calculating the IR spectrum of liquid water using APT exactly reproduces the one directly calculated from the DFT dipole moments sampled from an AIMD simulation [4]. Based on this discussion we would argue that the model responsible to describe the field-induced perturbation should also consider charge transfer and polarization effects. While such sophisticated models for liquid water exist, they would need to be fitted for different systems akin to training of the APTNN.

## References

[1] C. Schran, F. L. Thiemann, P. Rowe, E. A. Müller, O. Marsalek, and A. Michaelides. "Machine learning potentials for complex aqueous systems made simple". *Proc. Natl. Acad. Sci.* 118 (2021), e2110077118.

[2] M. Heyden, J. Sun, S. Funkner, G. Mathias, H. Forbert, M. Havenith, and D. Marx. "Dissecting the THz spectrum of liquid water from first principles via correlations in time and space". *Proc. Natl. Acad. Sci. USA* 107 (2010), pp. 12068–12073.

[3] O. Marsalek and T. E. Markland. "Quantum Dynamics and Spectroscopy of Ab Initio Liquid Water: The Interplay of Nuclear and Electronic Quantum Effects". *J. Phys. Chem. Lett.* 8 (2017), pp. 1545–1551.

[4] P. Schienbein. "Spectroscopy from machine learning by accurately representing the atomic polar tensor". *J. Chem. Theory Comput.* 19 (2023), pp. 705–712.

[5] M. Heyden, J. Sun, H. Forbert, G. Mathias, M. Havenith, and D. Marx. "Understanding the Origins of Dipolar Couplings and Correlated Motion in the Vibrational Spectrum of Water". *J. Phys. Chem. Lett.* 3 (2012), pp. 2135–2140.

[6] G. R. Medders and F. Paesani. "On the interplay of the potential energy and dipole moment surfaces in controlling the infrared activity of liquid water". *J. Chem. Phys.* 142 (2015), p. 212411.
